# Torsion of the whole ileum and hemorrhagic necrosis because of a giant Meckel’s diverticulum in a 9-month-old child

**DOI:** 10.1093/jscr/rjad336

**Published:** 2023-06-07

**Authors:** Qusai Mashlah, Ali M Zaher, Hajar Odah Bashi, Hanady Zwaraa, Husam Dalati

**Affiliations:** Department of Pediatric Surgery, Children Hospital, Damascus, Syria; Department of Pediatric Surgery, Children Hospital, Damascus, Syria; Department of Pediatric Surgery, Children Hospital, Damascus, Syria; Department of Pediatric Surgery, Children Hospital, Damascus, Syria; Department of Pediatric Surgery, Children Hospital, Damascus, Syria

**Keywords:** Intestinal torsion, Meckel’s diverticulum, Acute abdomen, Acute midgut volvulus

## Abstract

Intestinal ischemia caused by torsion of a freely hanging Meckel’s diverticulum (MD) resulting in the need for resection is an uncommon complication. We present an extraordinary case of a 9-month-old male with acute abdominal symptoms because of intestinal ischemia and necrosis that necessitated resection of the entire ileum. This was caused by torsion around a particularly large MD.

## INTRODUCTION

Meckel’s diverticulum (MD) is the most prevalent congenital anomaly of the small intestine, with a lifetime risk of 4–6% for developing complications. Clinical symptoms and complications may arise from small bowel obstruction, bleeding, inflammation, umbilical abnormalities or neoplasia. Intestinal obstruction is the most common surgical finding in symptomatic patients, accounting for 41% (with a range of 14–86%) of cases resulting from MD [1]. MD can lead to bowel obstruction via various mechanisms, such as intussusception, volvulus, vitelline bands/remnants, incarcerated Littre hernia and others [2]. Intussusception is slightly more common in children, and volvulus in adults.

## CASE REPORT

A 9-month-old male with sickle cell disease presented with severe vomiting, feeding intolerance, irritability and retention of stool and gasses for over 24 h. The patient did not have a history of rectal bleeding. On clinical examination, the patient exhibited mild abdominal tenderness. An ultrasound scan (US) showed a small to moderate amount of free fluid in Morrison’s sinus and splenic sinus, as well as between intestinal loops. No visible signs of intussusception were observed. The patient’s laboratory results showed a white blood cell count of 5400, hemoglobin level of 10.5, platelet count of 120 000, CRP of 6, Na of 138 and K of 5.4.

**Figure 1:**
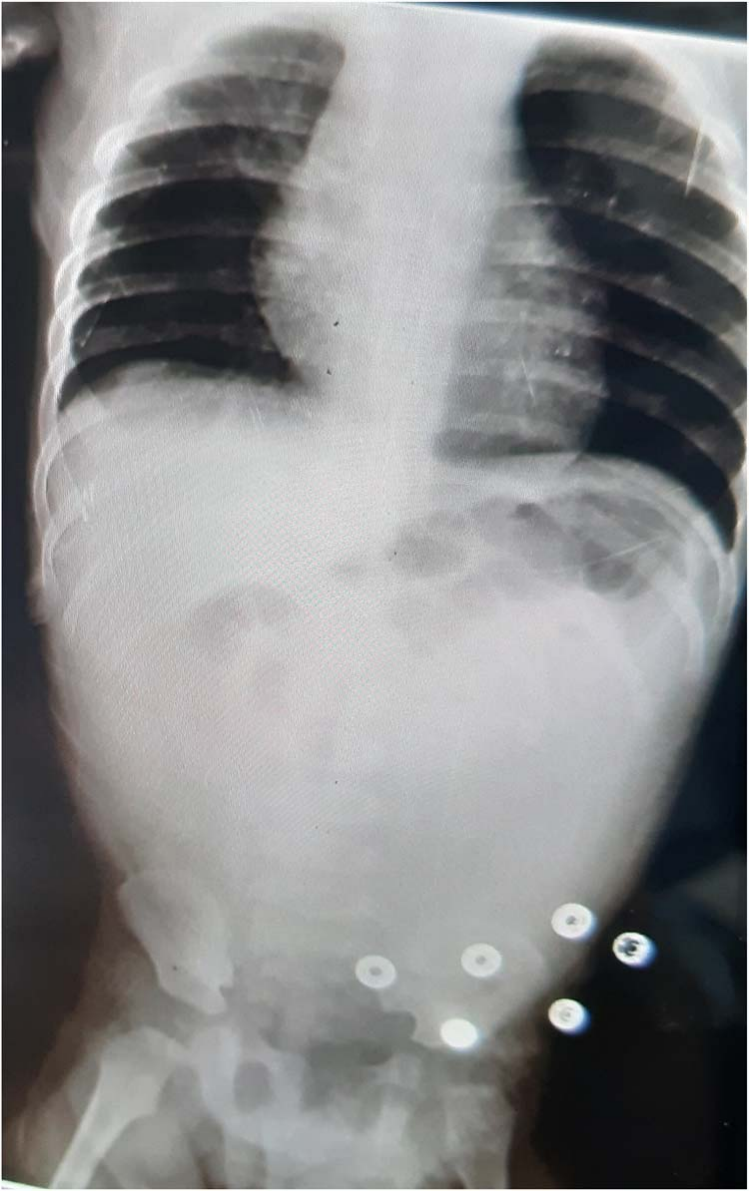
AXR: no gas liquid levels—no free gas in the abdomen—dilation of intestinal loops and lack of gas in the abdomen and pelvis.

An abdominal X-ray revealed no gas liquid levels or free gas in the abdomen. Intestinal loops were dilated, and there was a lack of gas in the abdomen and pelvis. Twelve hours later, the patient developed severe abdominal pain with generalized abdominal guarding. The hemoglobin level dropped to 6.8, necessitating an emergency exploratory laparotomy (Fig. 1).

**Figure 2:**
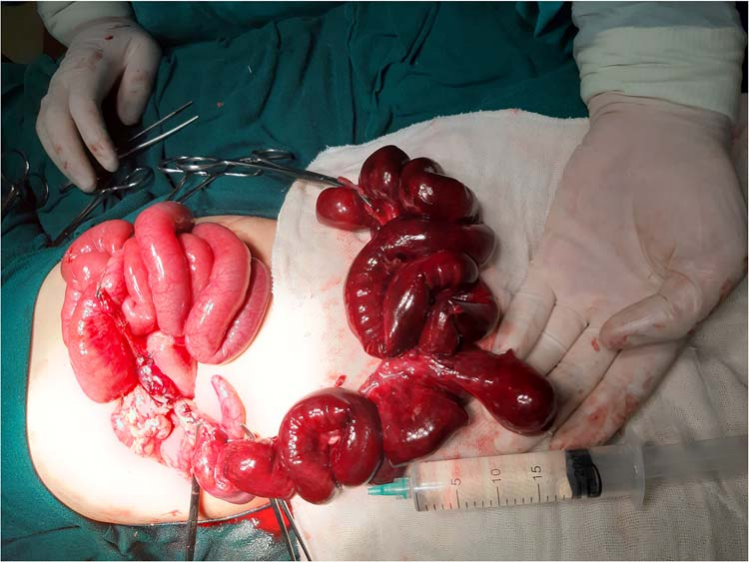
Image during resection showing giant MD and necrotic small intestine.

During surgical investigation, a small bowel torsion was discovered extending from the ileocecal junction to 120 cm of ileum, which included a broad-based, inflamed giant MD. Attempts to reverse the torsion and warm the intestines for 25 min did not improve perfusion. As a result, the damaged intestine, cecum and appendix were amputated (Fig. 2), and an end-to-end two-layer ileocolic anastomosis was performed.

The patient began oral feeding on the 5th day post-surgery with good tolerance and was discharged on the 7th day. Histopathological examination revealed hemorrhagic necrosis in the intestine up to the ileum margins, consistent with torsion of the intestine with diverticular formation showing hemorrhagic necrosis. The appendix showed nonspecific follicular hyperplasia with bleeding.

## DISCUSSION

Approximately 4% of individuals with MD will exhibit symptoms. The predominant causes of symptomatic Meckel’s are intestinal obstruction, which can occur through various mechanisms such as intussusception or volvulus. Volvulus can arise from fibrous or vascular remnants of the omphalodiverticular tract (extending from the tip of the diverticulum to the abdominal wall). Giant Meckel diverticula or vitelline cysts may also cause volvulus. If the volvulus causes ischemia, the patient may exhibit signs of peritonitis and may present in severe cases such as the one being discussed. Torsion of an MD resulting in intestinal ischemia that requires resection is a rare complication [3]. In rare instances, necrosis of the intestine because of MD with no identifiable point of fixation has been reported [1, 4–7].

Acute midgut volvulus (MGV) is considered the most severe form of volvulus and has been associated with a fourfold increase in mortality risk in comparison to segmental volvulus. The overall mortality rate for MGV ranges from 2.2 to 16%. Approximately 75% of MGV cases occur in the 1st year of life, and most cases occur during the neonatal period. The incidence of bowel necrosis with MGV in other reports was found to range from 2.9 to 48%. Heart rate; mean arterial pressure; pH; and serum CRP, sodium and albumin levels are independent predictors for intestinal ischemia and necrosis in patients with MGV [8]. The presence of superior mesenteric artery collapse, ascites and a large intestinal twist on US imaging were significant predictors of intestinal ischemic changes [9].

When the ileum adjacent to the diverticulum is ischemic, resection of the affected bowel with an ileo-ileostomy is preferred [5]. In patients with intestinal obstruction, delaying surgery for more than 36 h increases the mortality rate from 8 to 25% [10].

## CONCLUSION

The occurrence of intestinal ischemia necessitating resection because of MD is an infrequent yet life-threatening complication. Prompt diagnosis and timely surgical intervention are crucial in preventing necrosis of the midgut, mortality and short bowel syndrome resulting from extensive small bowel resection. This case reinforces the possibility of bowel torsion in MD without the presence of a fibrous band or adhesion. Furthermore, laboratory analyses and abdominal imaging with ultrasound may not reveal any evidence of ischemia or necrosis in the small bowel.

## Data Availability

All data underlying the results are available as part of the article and no additional source data are required.

## References

[1] Keese D, Rolle U, Gfroerer S, Fiegel H. Symptomatic Meckel’s diverticulum in pediatric patients-case reports and systematic review of the literature. Front Pediatr 2019;7. 10.3389/fped.2019.00267 PMC6606722 31294008

[2] Sran HS, Narula IS, Dandia SD, Pendse AK. Meckel’s diverticulum causing intestinal obstruction. Am J Gastroenterol 2001;96:116–9. 4761340

[3] Halliday J, Jamieson R, Gillies T. Meckels diverticulum and intestinal ischaemia. J Surg Case Rep 2011;2011:5. 10.1093/jscr/2011.1.5 PMC3649195 24950543

[4] Musteata L, Geraldo RF, Ndasu Matendo H, Reitz A, Krasovski V, Nitu V, et al. Meckel diverticulum causing intestinal volvulus. Case Rep Surg 2020;2020:1–3. 10.1155/2020/8872668 PMC7533013 33062366

[5] Hung ND, Tuan TA, Sy TV, Thinh VD, Ngoc VTN, Nga VT, et al. Torsion of ileum due to giant Meckel’s diverticulum - a case report. Open Access Maced J Med Sci 2019;7:4432–4. 32215108 10.3889/oamjms.2019.876 PMC7084003

[6] Omole PW, Mujinga DT, Lubosha NA, Mujinga IMW, Ntanga DI. Occlusion intestinale sur diverticule de Meckel: à propos d’un cas. Pan Afr Med J 2019;32:117. 10.11604/pamj.2019.32.117.16523 PMC6561005 31223407

[7] Kirmizi S, Kirmizi DA, Karagul R, Tolan K. Giant Meckel’s diverticulum torsion that mimics adnexal pathology. Int J Surg Case Rep 2016;24:139–41. 27261632 10.1016/j.ijscr.2016.05.033 PMC4898912

[8] Guan X, Wang Z, He Q, Lv J, Yu J, Zhong W. Nomogram for estimating the risks of intestinal ischemia and necrosis in neonates with midgut volvulus: a retrospective study. Front Pediatr 2022;10:888594. 10.3389/fped.2022.888594 PMC9251320 35795333

[9] Hosokawa T, Hosokawa M, Tanami Y, Sato Y, Ishimaru T, Tanaka Y, et al. Use of ultrasound findings to predict bowel ischemic changes in pediatric patients with intestinal volvulus. J Ultrasound Med 2020;39:683–92. 31642550 10.1002/jum.15145

[10] You JS, Chung SP, Park YS, Yu JS, Park YA. A case of strangulated small bowel obstruction caused by Meckel’s diverticulum in an adult. J Emerg Med 2007;33:133–5. 17692763 10.1016/j.jemermed.2007.01.008

